# The value of lncRNAs as prognostic biomarkers on clinical outcomes in osteosarcoma: a meta-analysis

**DOI:** 10.1186/s12885-021-07882-w

**Published:** 2021-02-27

**Authors:** Wenchao Zhang, Xiaolei Ren, Lin Qi, Chenghao Zhang, Chao Tu, Zhihong Li

**Affiliations:** 1 Department of Orthopedics, The Second Xiangya Hospital, Central South University, Changsha, 410011 Hunan People’s Republic of China; 2 Hunan Key Laboratory of Tumor Models and Individualized Medicine, The Second Xiangya Hospital, Central South University, Changsha, Hunan People’s Republic of China

**Keywords:** LncRNAs, Osteosarcoma, Meta-analysis, Prognosis

## Abstract

**Background:**

In recent years, emerging studies have demonstrated critical functions and potential clinical applications of long non-coding RNA (lncRNA) in osteosarcoma. To further validate the prognostic value of multiple lncRNAs, we have conducted this updated meta-analysis.

**Methods:**

Literature retrieval was conducted by searching PubMed, Web of Science and the Cochrane Library (last update by October 2, 2019). A meta-analysis was performed to explore association between lncRNAs expression and overall survival (OS) of osteosarcoma patients. Relationships between lncRNAs expression and other clinicopathological features were also analyzed respectively.

**Results:**

Overall, 4351 patients from 62 studies were included in this meta-analysis and 25 lncRNAs were identified. Pooled analyses showed that high expression of 14 lncRNAs connoted worse OS, while two lncRNAs were associated with positive outcome. Further, analysis toward osteosarcoma clinicopathologic features demonstrated that overexpression of TUG1 and XIST indicated poor clinical parameters of patients.

**Conclusions:**

This meta-analysis has elucidated the prognostic potential of 16 lncRNAs in human osteosarcoma. Evidently, desperate expression and functional targets of these lncRNAs offer new approaches for prognosis and therapy of osteosarcoma.

**Supplementary Information:**

The online version contains supplementary material available at 10.1186/s12885-021-07882-w.

## Background

Osteosarcoma is the most common primary bone malignancy with an annual incidence of 3.1 per million [1]. Despite various treatments, such as chemotherapy, radiotherapy, surgery and targeted therapy, have been used for osteosarcoma, the prognosis remains poor [2, 3]. Of note, the 5-year survival rate for children and adults with non-metastatic osteosarcoma is 71.8%, while for patients with metastatic osteosarcoma dramatically decrease to 30.4% [4, 5]. Therefore, identification of new prognostic or therapeutic hallmarks are in urgent need to improve current situation. In fact, numerous studies have been conducted upon this issue in recent years, and some have shed light on the roles of multiple molecules, including RNAs, regulatory proteins, etc. [6–8]

With advancement of next-generation sequencing technologies, several kinds of non-coding RNAs (ncRNAs) have been discovered, such as the miRNA, siRNA, snoRNA, piRNA and lncRNA. LncRNAs, a cluster of non-coding RNA with more than 200 nucleotides, show no potential of protein coding but exert crucial functions in maintenance of the cellular homeostasis [9]. Mechanisms of lncRNAs in biological processes contain chromatin modifications, transcriptional modifications and post-transcriptional modifications that regulate the expression and features of other genes [10]. They have been elucidated to play critical roles in the development of various diseases, especially tumors [11]. Gouri et al. have reviewed the roles of lncRNAs in pancreatic ductal adenocarcinoma in which they demonstrated that lncRNAs closely associated with the tumorigenesis, partially through dysregulating the KRAS pathway. And it was noticed that the expression level of multiple lncRNAs were altered in tissue, plasma or serum specimens of pancreatic cancer patients, which support the idea that lncRNAs may serve as therapeutic biomarker for pancreatic ductal adenocarcinoma [12]. Moreover, researchers have demonstrated functional mechanisms of lncRNAs in regulating multiple physiological and pathophysiological processes by interacting with other intrinsic molecules [13]. Notably, roles of lncRNAs in progression, prognosis and metastasis of osteosarcoma have been broadly identified [14]. And circulating lncRNAs showed significant potential in osteosarcoma prognosis [15]. To further demonstrate the roles and prognostic potential of lncRNAs in osteosarcoma, we have conducted this meta-analysis.

## Methods

### Literature search strategy

Two independent researchers retrieved the published literature from database of Pubmed, Web of Science and Cochrane Library at the same time. Search terms used were the following: (osteosarcoma OR “osteogenic sarcoma”) AND (“long non-coding RNA” OR lncRNA OR “LINC RNA” OR “Long ncRNAs”). The last research time was October 2, 2019.

### Selection criteria

A total of 550 articles were initially identified after removal of duplication. Two independent researchers (Wenchao Zhang and Xiaolei Ren) reviewed the title, abstract and full-text of all included articles. Articles that met the following criteria were included: (1) Research topic related to the lncRNAs expression and osteosarcoma prognosis; (2) the survival outcome was available in OS form and shown in table, Kaplan-Meier curve or HR value; (3) patients were divided into two groups based on the expression of lncRNAs (high versus low). The exclusive criteria were: (1) the review, case report, conference abstract, letters, sequencing data, bioinformatics analysis, retreated articles and meta-analysis; (2) survival data was obtained from online database such as the TCGA; (3) when more than one study reported on the same patient cohort, only the most recent one was included. Disagreements between the two reviewers were discussed to reach an consensus.

### Data extraction and quality assessment

All articles were reviewed by two independent well-trained investigators to retrieve available data. The following information was listed for all articles: Name of first author, publication year, country where study conducted, detection method, sample number, lncRNA type, tumor stage, follow-up time, cut-off value, survival data (multivariate analysis was prioritized if both the univariate and multivariate analysis were provided), outcome measure, Hazard ratio (HR) of lncRNAs expression for OS and the corresponding 95% CI. If HR and 95% CI were not directly provided while a Kaplan-Meier curve was available, we retrieved the HR and 95% CI by using Engauge Digitizer version 4.1 and Tierney’s method as previously described [16]. Then, the quality of the included studies was assessed by two independent reviewers (Wenchao Zhang and Xiaolei Ren) by following the Newcastle-Ottawa Scale (NOS) [17].

### Statistical analysis

Stata 12.0 (Stata Corporation, TX, USA) and Review Manager 5.3 (The Cochrane Collaboration, 2014.) were used in this meta-analysis to pool the HR and its 95% CI. Q-test was applied to evaluate the heterogeneity among the studies [18]. If the heterogeneity was substantial (I^2^ > 50%, *P* < 0.05), the random effect model would be adopted, otherwise the fixed effect model would be used. A pooled HR > 1 connoted a poor prognosis in patients with lncRNAs overexpression, while a pooled HR < 1 supported a better prognosis. *P* < 0.05 was considered statistically significant. LncRNAs that have been studied in more than two articles were included to further analyze the clinical parameters, aiming to figure out the association between clinical parameters and lncRNAs expression. Publication bias was measured by Begg’s and Egger’s tests [19].

## Results

### Characteristics of included studies

**Fig. 1:**
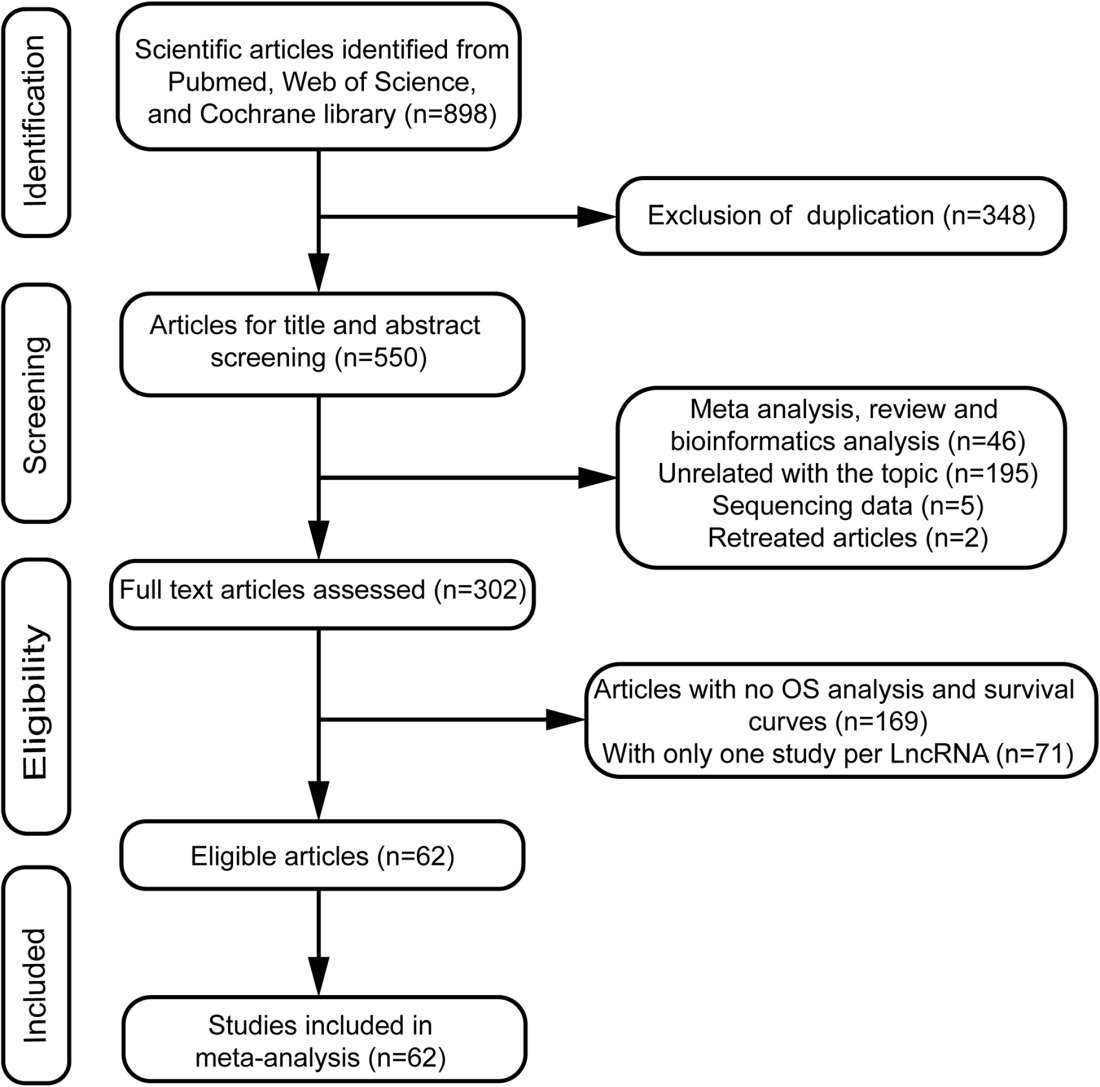
Study flow chart

Overall, 62 articles comprising 4351 patients were recruited in our study after selection by following the criteria. The study filtrating process was shown in Fig. 1. Among the included 62 articles, 25 lncRNAs were identified and only four have been studied in more than three articles. The most extensively studied lncRNA was Metastasis-associated lung adenocarcinoma transcript 1 (MALAT1), which was shown in eight articles, followed by Taurine up-regulated gene 1 (TUG1), X-inactive specific transcript (XIST) and Nuclear enriched abundant transcript 1 (NEAT1). Almost all of the studies were conducted in China while only one study originated from Brazil. All the articles were published between 2015 and 2019, mostly in 2018 and 2019. The sample number in the selected studies ranged from 30 to 204. All studies used quantitative real-time polymerase chain reaction (qRT-PCR) to measure the expression of lncRNAs, and tissue was the most widely used sample. The cut-off values of lncRNAs expression varied among studies, mainly including median, optimal or mean value. The specific information was shown in Table 1. The quality evaluation showed that NOS scores of all the included studies were greater than 5 (Additional Figures S1 and S2).

**Table 1:** The main characteristics of the included studies in the meta-analysis

ID	Study	country	LncRNA name	Sample number	Tumor stage	Distant metastasis	Follow-up (months)	Detected sample	Detection method	Cut-off value	Survival analysis	Outcome measure	HR (95% CI)
1	Ju L (2016) [20]	China	BCAR4	168	IIA-IIB/III	48	70	tissue	qRT–PCR	median	Multivariate analysis	OS	3.22 (0.89, 7.88)
2	Chen F (2016) [21]	China	BCAR4	60	I-III	13	60	tissue	qRT–PCR	median	N/A	OS/RFS	1.83 (0.68, 4.96)
3	Ruan R (2018) [22]	China	CCAT2	50	IA-III	16	70	tissue	qRT–PCR	median	N/A	OS	1.30 (0.60, 2.84)
4	Yan L (2018) [23]	China	CCAT2	40	N/A	N/A	60	tissue	qRT–PCR	N/A	N/A	OS/RFS	3.57 (1.25, 5.31)
5	Jiang N (2017) [24]	China	DANCR	34	N/A	18	60	tissue	qRT–PCR	N/A	Multivariate analysis	OS/DFS	1.08 (1.23, 5.79)
6	Wang Y (2018) [25]	China	DANCR	95	I-III	58	60	tissue	qRT–PCR	Optimal	N/A	OS	2.4 (0.85, 3.25)
7	Fei D (2018) [26]	China	FER1L4	48	I-IV	10	60	tissue	qRT–PCR	median	N/A	OS/PFS	0.45 (0.17, 0.97)
8	Chen Z (2018) [27]	China	FER1L4	73	I-IV	25	60	tissue	qRT–PCR	N/A	N/A	OS/PFS	0.43 (0.05, 0.86)
9	Ren Z (2019) [28]	China	FOXD2-AS1	35	I-III	N/A	60	tissue	qRT–PCR	N/A	N/A	OS	1.66 (0.47, 5.82)
10	Zhang H (2019) [29]	China	FOXD2-AS1	40	N/A	N/A	70	tissue	qRT–PCR	N/A	N/A	OS	1.55 (0.3, 7.86)
11	Cai L (2017) [30]	China	HNF1A-AS1	72	IIA-III	42	<60	tissue	qRT–PCR	median	Multivariate analysis	OS	2.63 (1.55, 5.65)
12	Zhao H (2016) [31]	China	HNF1A-AS1	43	I-III	11	60	tissue	qRT–PCR	median	Multivariate analysis	OS	2.64 (1.39, 7.42)
13	Cao K (2019) [32]	China	HOXA11-AS	61	I-III	17	60	tissue	qRT–PCR	median	N/A	OS	1.5 (0.43, 5.26)
14	Cui M (2017) [33]	China	HOXA11-AS	51	I-III	13	60	tissue	qRT–PCR	median	N/A	OS	3.22 (0.31, 33.42)
15	Gu W (2018) [34]	China	HOXD-AS1	43	I-III	N/A	60	tissue	qRT–PCR	N/A	N/A	OS	1.39 (0.28, 7.02)
16	Qu Y (2018) [35]	China	HOXD-AS1	46	I-IV	11	60	tissue	qRT–PCR	median	N/A	OS	1.63 (1.19, 2.96)
17	Maciel Uzan (2016) [36]	Brazil	HULC	33	I-IV	10	100	tissue	qRT–PCR	optimal	Multivariate analysis	OS/EFS	22.01 (2.26, 16.13)
18	Sun X (2015) [37]	China	HULC	78	IIA-III	21	60	tissue	qRT–PCR	median	Multivariate analysis	OS	2.28 (1.48, 5.43)
19	He W (2019) [38]	China	LSINCT5	124	I-III	22	72	tissue	qRT–PCR	median	Multivariate analysis	OS	1.68 (1.02, 2.76)
20	Kong D (2018) [39]	China	LSINCT5	42	I-IV	18	60	tissue	qRT–PCR	N/A	N/A	OS/DFS	1.39 (1.13, 5.68)
21	Chen Y (2018) [40]	China	MALAT1	68	N/A	N/A	60	tissue	qRT–PCR	N/A	Multivariate analysis	OS/DFS	1.73 (1.10, 2.54)
22	Gao K (2016) [41]	China	MALAT1	162	IIA-III	44	70	tissue	qRT–PCR	median	Multivariate analysis	OS	2.8 (1.76, 7.84)
23	Huo Y (2017) [42]	China	MALAT1	68	I-IV	22	80	serum	qRT–PCR	median	N/A	OS/PFS	3.33 (1.43, 4.91)
24	Li Q (2017) [43]	China	MALAT1	64	I-IV	33	60	tissue	qRT–PCR	optimal	Multivariate analysis	OS	2.22 (0.3, 16.44)
25	Sun Y (2018) [44]	China	MALAT1	42	I-III	20	60	tissue	qRT–PCR	N/A	N/A	OS	2.2 (1.15, 4.21)
26	Sun Z (2019) [45]	China	MALAT1	76	I-III	46	120	tissue	qRT–PCR	N/A	N/A	OS	2.51 (0.79, 7.99)
27	Wang J (2017) [46]	China	MALAT1	70	N/A	N/A	<60	tissue	qRT–PCR	N/A	N/A	OS	2.34 (0.55, 9.92)
28	Wang Y (2017b) [47]	China	MALAT1	55	I-III	31	60	tissue	qRT–PCR	median	N/A	OS	1.01 (0.25, 4.03)
29	Shen B (2019) [48]	China	MEG3	204	I-III	80	50	tissue	qRT–PCR	median	Multivariate analysis	OS	0.56 (0.36, 0.87)
30	Tian Z (2015) [49]	China	MEG3	64	I-III	17	60	tissue	qRT–PCR	median	Multivariate analysis	OS	0.45 (0.11, 1.81)
31	Ji S (2019) [50]	China	NEAT1	72	N/A	N/A	160	tissue	qRT–PCR	median	N/A	OS	2.09 (0.84, 5.22)
32	Li Y (2018) [51]	China	NEAT1	76	I-III	38	60	tissue	qRT–PCR	median	N/A	OS	1.88 (0.47, 7.49)
33	Tan H (2019) [52]	China	NEAT1	47	I-III	11	50	tissue	qRT–PCR	median	N/A	OS	1.84 (0.61, 5.57)
34	Zhu K (2019) [53]	China	OIP5-AS1	80	N/A	N/A	60	tissue	qRT–PCR	N/A	N/A	OS	1.48 (0.42, 5.15)
35	Dai J (2018) [54]	China	OIP5-AS1	48	I-III	N/A	60	tissue	qRT–PCR	median	N/A	OS	1.77 (1.17, 2.94)
36	Zhang C (2016) [55]	China	ODRUL	60	N/A	12	80	tissue	qRT–PCR	median	N/A	OS	1.21 (0.36, 4.06)
37	Zhu K (2017) [56]	China	ODRUL	80	N/A	48	100	tissue	qRT–PCR	N/A	N/A	OS	2.35 (1.08, 5.15)
38	Huang J (2018) [57]	China	PCAT1	62	I-III	35	60	tissue	qRT–PCR	N/A	Multivariate analysis	OS/PFS	1.53 (1.37, 2.92)
39	Zhang X (2018) [58]	China	PCAT1	30	I-III	17	60	tissue	qRT–PCR	mean	Multivariate analysis	OS	4.01 (1.56, 4.57)
40	Song J (2017) [59]	China	PVT1	46	I-III	N/A	70	tissue	qRT–PCR	mean	N/A	OS	1.63 (0.05, 5.39)
41	Zhou Q (2016) [60]	China	PVT1	53	N/A	13	60	tissue	qRT–PCR	N/A	N/A	OS	1.70 (0.66, 4.37)
42	Zhou B (2018) [61]	China	SNHG12	64	N/A	N/A	80	tissue	qRT–PCR	N/A	N/A	OS	2.10 (0.66, 6.64)
43	Zhou S (2018) [62]	China	SNHG12	31	I-III	21	60	tissue	qRT–PCR	mean	N/A	OS	1.52 (1.00, 18.48)
44	Liao S (2019) [63]	China	SNHG16	96	I-III	51	60	tissue	qRT–PCR	mean	Multivariate analysis	OS	1.58 (0.65, 3.86)
45	Wang X (2019) [64]	China	SNHG16	65	I-III	28	60	tissue	qRT–PCR	median	N/A	OS	2.62 (0.82, 8.36)
46	Wang W (2018) [65]	China	SNHG20	32	I-III	N/A	60	tissue	qRT–PCR	median	N/A	OS	1.94 (1.19, 3.17)
47	Zhang J (2018) [66]	China	SNHG20	140	I-III	24	72	tissue	qRT–PCR	N/A	Multivariate analysis	OS	2.05 (0.53, 7.92)
48	Chen X (2019) [67]	China	TP73-AS1	132	I-III	22	72	tissue	qRT–PCR	N/A	Multivariate analysis	OS	1.89 (1.15, 3.13)
49	Yang G (2018) [68]	China	TP73-AS1	46	I-III	11	50	tissue	qRT–PCR	mean	N/A	OS	1.98 (0.61, 6.28)
50	Ma B (2016) [69]	China	TUG1	76	I-III	36	60	tissue	qRT–PCR	optomal	Multivariate analysis	OS/PFS	2.77 (1.29, 5.98)
51	Sheng K (2019) [70]	China	TUG1	40	N/A	26	60	plasma	qRT–PCR	median	N/A	OS	1.59 (1.36, 1.97)
52	Wang Q (2018) [71]	China	TUG1	94	IIA -III	26	70	tissue	qRT–PCR	median	Multivariate3analysis	OS	1.10 (0.96, 15.35)
53	Wang Y (2017a) [72]	China	TUG1	44	I-III	28	120	tissue	qRT–PCR	N/A	N/A	OS	2.12 (1.44, 3.67)
54	Yu X (2019) [73]	China	TUG1	40	I-III	11	60	tissue	qRT–PCR	N/A	N/A	OS/RFS	1.44 (1.15, 2.54)
55	Li W (2016) [74]	China	UCA1	135	I-III	34	60	tissue	qRT–PCR	median	Multivariate analysis	OS	2.19 (0.87, 5.55)
56	Wen J (2017) [75]	China	UCA1	151	IIA -III	79	60	tissue	qRT–PCR	optimal	Multivariate analysis	OS	2.52 (1.34, 4.83)
57	Li G (2017) [76]	China	XIST	145	I-IV	44	60	tissue	qRT–PCR	N/A	N/A	OS	1.75 (1.00, 3.06)
58	Wang W (2019) [77]	China	XIST	64	IA -III	15	70	tissue	qRT–PCR	median	N/A	OS	1.92 (1.25, 3.29)
59	Yang C (2018) [78]	China	XIST	40	I-III	N/A	60	tissue	qRT–PCR	N/A	N/A	OS	3.07 (1.84, 11.15)
60	Zhang R (2017) [79]	China	XIST	50	N/A	26	60	tissue	qRT–PCR	N/A	N/A	OS	1.59 (1.38, 2.88)
61	Li N (2017) [80]	China	ZFAS1	53	I-III	16	60	tissue	qRT–PCR	median	Multivariate analysis	OS/RFS	1.14 (0.23, 5.61)
62	Liu G (2017) [81]	China	ZFAS1	50	N/A	N/A	30	tissue	qRT–PCR	median	N/A	OS	1.59 (0.64, 3.95)

DFS disease-free survival, PFS progression-free survival, RFS recurrence-free survival, OS overall survival, N/A not available

### Overexpression of lncRNAs indicate different prognosis of osteosarcoma

In this meta-analysis, 25 lncRNAs were analyzed individually. The result showed that overexpression of 14 lncRNAs were associated with poor prognosis while two indicated a positive outcome. The overexpression of the rest nine lncRNAs were independent of osteosarcoma prognosis (Table 2).

**Table 2:** meta-analysis results of 25 lncRNAs

LncRNA name	No. of patients	No. of studies	HR(95%CI)-model	*P* value	heterogeneity	
					I^2^	*P*
BCAR4	228	2	2.36 (1.13, 4.93)-fixed	0.022	0.0%	0.453
CCAT2	90	2	2.18 (0.81, 5.86)-random	0.123	71.2%	0.062
DANCR	129	2	1.65 (0.76, 3.60)-random	0.209	57.1%	0.127
FER1L4	121	2	0.44 (0.16, 0.72)-fixed	0.032	0.0%	0.945
FOXD2-AS1	75	2	1.62 (0.60, 4.38)-fixed	0.344	0.0%	0.948
HNF1A-AS1	115	2	2.63 (1.58, 4.39)-fixed	0.000	0.0%	0.994
HOXA11-AS	112	2	1.78 (0.59, 3.36)-fixed	0.307	0.0%	0.573
HOXD-AS1	89	2	1.61 (1.04, 2.50)-fixed	0.033	0.0%	0.852
HULC	111	2	5.38 (0.62, 46.50)-random	0.126	71.5%	0.061
LSINCT5	166	2	1.59 (1.04, 2.44)-fixed	0.031	0.0%	0.695
MALAT1	605	8	2.15 (1.67, 2.76)-fixed	0.000	0.0%	0.676
MEG3	268	2	0.55 (0.36, 0.84)-fixed	0.005	0.0%	0.770
NEAT1	195	3	1.96 (1.05, 3.68)-fixed	0.035	0.0%	0.983
ODRUL	140	2	1.73 (1.12, 2.67)-fixed	0.013	0.0%	0.793
OIP5-AS1	128	2	1.93 (1.00, 3.73)-fixed	0.049	0.0%	0.367
PCAT1	92	2	2.43 (1.95, 6.24)-random	0.065	87.9%	0.004
PVT1	99	2	1.69 (0.70, 4.06)-fixed	0.241	0.0%	0.974
SNHG12	95	2	1.85 (0.75, 4.58)-fixed	0.181	0.0%	0.733
SNHG16	161	2	1.91 (0.94, 3.86)-fixed	0.074	0.0%	0.498
SNHG20	172	2	1.95 (1.23, 3.09)-fixed	0.004	0.0%	0.940
TP73	178	2	1.90 (1.20, 3.02)-fixed	0.006	0.0%	0.943
TUG1	294	5	1.64 (1.42, 1.92)-fixed	0.000	0.0%	0.435
UCA1	286	2	2.41 (1.42, 4.07)-fixed	0.001	0.0%	0.809
XIST	299	4	1.79 (1.40, 2.30)-fixed	0.000	0.0%	0.601
ZFAS1	103	2	1.47 (0.66, 3.23)-fixed	0.343	0.0%	0.723

CI confidence interval, HR hazard ratio

**Fig. 2:**
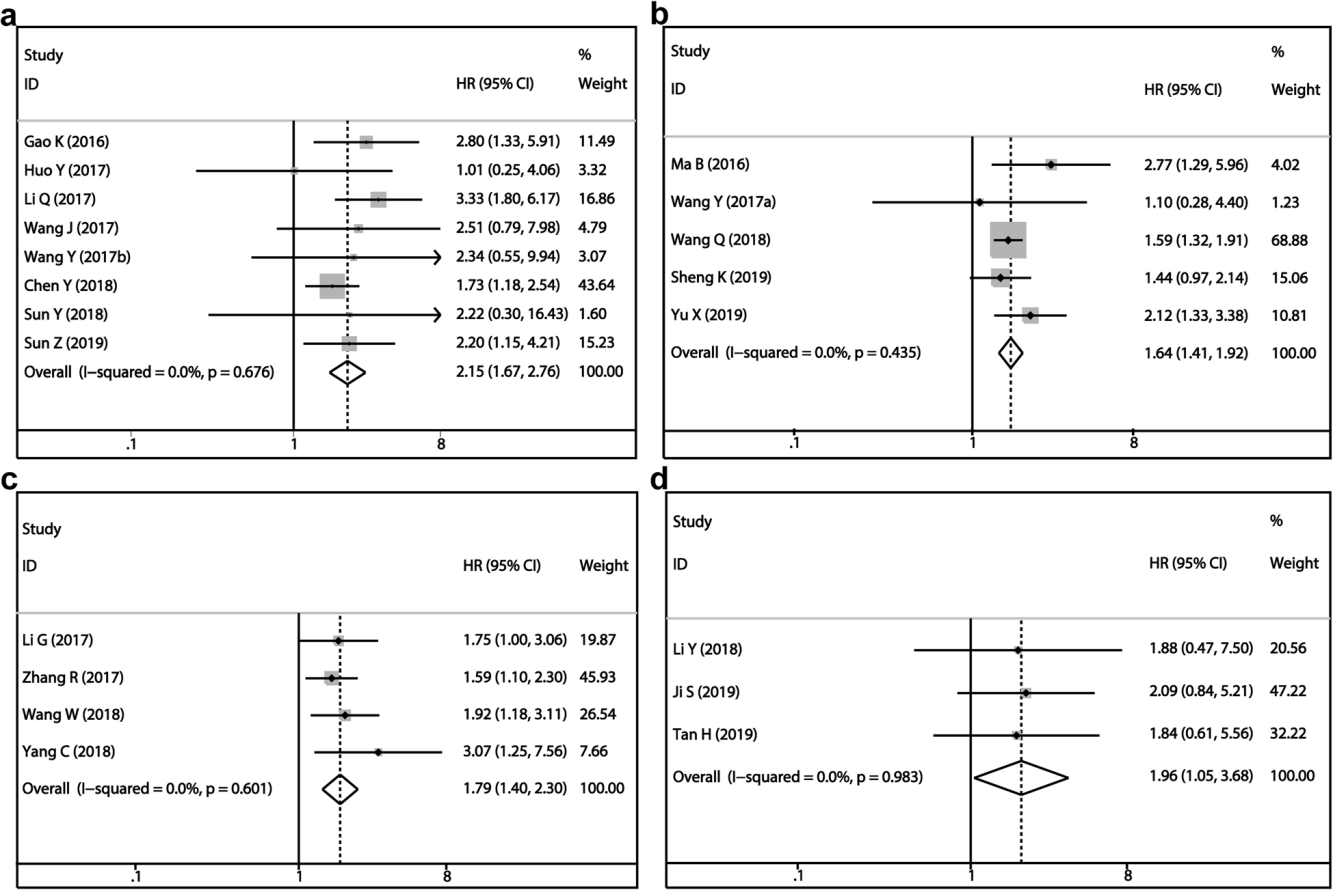
Forest plot of overall survival for four most studied lncRNAs: **a** MALAT1, **b** TUG1, **c** XIST, **d** NEAT1

More attention was paid to the four most studied lncRNAs among all included studies, the MALAT1, TUG1, XIST and NEAT1. For MALAT1, eight articles included 605 patients were pooled. Overexpression of MALAT1 was a risk factor of osteosarcoma (HR = 2.15, 95%CI: 1.67–2.76, *P* < 0.001, Fig. 2a). We noticed that in one of these eight studies, the detected sample was serum rather than tissue. So, we analyzed the remaining seven studies after eliminating this one, and the predicted tendence of MALAT1 in osteosarcoma was not altered (HR = 2.20, 95%CI: 1.70–2.85, *P* < 0.001). Since no heterogeneity among studies was noted, we did not perform subgroup analysis (I^2^ = 0.0%, *P* = 0.676). Then, five studies focused on TUG1 containing 294 patients were analyzed, which showed that overexpression of TUG1 was associated with unfavorable clinical outcome of osteosarcoma patients (HR = 2.41, 95%CI: 1.42–4.07, *P* = 0.001, Fig. 2b). There was also a study that used plasma instead of tissue as the detected sample. Thus, we did another analysis without this study. The result showed the negative prediction role of TUG1 as well. (HR = 1.68, 95%CI: 1.43–1.99, *P* = 0.001). A negative association between XIST expression and OS of osteosarcoma patients was noticed (HR = 1.79, 95%CI: 1.40–2.30, *P* < 0.001, Fig. 2c) based on the analysis of four researches containing 299 patients. Finally, three studies incorporating 199 patients were analyzed to explore the association between NEAT1 expression and OS. It proved that the high expression of NEAT1 foreboded poor prognosis. (HR = 1.96, 95%CI: 1.05–3.68, *P* = 0.035, Fig. 2d).

### LncRNAs expression and osteosarcoma clinicopathologic features

Furthermore, the clinicopathologic features, including age, gender, clinical stage, tumor size and distant metastasis, were analyzed. We mainly focused on the MALAT1, TUG1 and XIST, lncRNAs that owned available data we needed in more than three articles. There were no significant differences in MALAT1 expression detected in different age (HR = 0.73, 95%CI: 0.43–1.24, *P* = 0.240), gender (HR = 0.73, 95%CI: 0.44–1.20, *P* = 0.210), clinical stage (HR = 1.48, 95%CI: 0.27–7.94, *P* = 0.650), tumor size (HR = 0.95, 95%CI: 0.50–1.81, *P* = 0.890) and distant metastasis (HR = 1.98, 95%CI: 0.32–12.05, *P* = 0.40). However, the distinction of TUG1 expression was observed in different clinical stage (HR = 4.66, 95%CI: 2.47–8.79, *P* < 0.001), tumor size (HR = 4.07, 95%CI: 2.33–7.12, *P* < 0.001) and distant metastasis (HR = 3.53, 95%CI: 1.20–10.41, *P* = 0.020). Osteosarcoma tissue derived from patients with higher clinical stage, larger tumor size and distant metastasis expressed high TUG1. Differences also have been found in XIST expression upon the clinical stage and metastasis. High clinical stage (HR = 3.92, 95%CI: 2.31–6.66, *P* < 0.001) and metastasis (HR = 3.15, 95%CI: 1.64–6.05, *P* < 0.001) were associated with high expression of XIST in tumor tissue. More detailed information was shown in Table 3.

**Table 3:** Analysis of clinical features

Outcome	No. of Studies	No. of Participants	OR (95% CI)	*P* value	Model	Heterogeneity Chi^2^, *P*-value, *I*^*2*^
**MALAT1**						
Age	3	259	0.73 (0.43, 1.24)	0.24	Fixed	0.36, 0.84, 0%
Gender	3	259	0.73 (0.44, 1.20)	0.21	Fixed	0.16, 0.92, 0%
Clinical stage	3	259	1.48 (0.27, 7.94)	0.65	Random	15.91, 0.0004, 87%
Tumor size	3	259	0.95 (0.50, 1.81)	0.89	Random	10.77, 0.005, 81%
Distant metastasis	3	259	1.98 (0.32, 12.05)	0.46	Random	16.26, 0.0003, 88%
**TUG1**						
Age	5	294	1.2 (0.48, 3.02)	0.28	Fixed	1.97, 0.74, 0%
Gender	5	294	1.02 (0.62, 1.65)	0.95	Fixed	2.97, 0.56, 0%
Clinical stage	4	254	4.66 (2.47, 8.79)	<0.00001	Fixed	0.45, 0.93, 0%
Tumor size	4	254	4.07 (2.33, 7.12)	<0.00001	Fixed	2.96, 0.4, 0%
Distant metastasis	5	294	3.53 (1.20, 10.41)	0.02	Random	13.52, 0.009, 70%
**XIST**						
Age	2	209	1.18 (0.44, 3.15)	0.74	Random	2.55, 0.11, 61%
Gender	3	249	0.91 (0.56, 1.50)	0.72	Fixed	1.56, 0.46, 0%
Clinical stage	3	249	3.92 (2.31, 6.66)	<0.00001	Fixed	0.92, 0.63, 0%
Tumor size	3	249	1.15 (0.41, 3.23)	0.80	Random	6.74, 0.03, 70%
Distant metastasis	2	209	3.10 (1.61, 5.95)	0.0007	Fixed	0.58, 0.45, 0%

*CI* confidence interval, *OR* odds ratio

### Sensitivity analysis

**Fig. 3:**
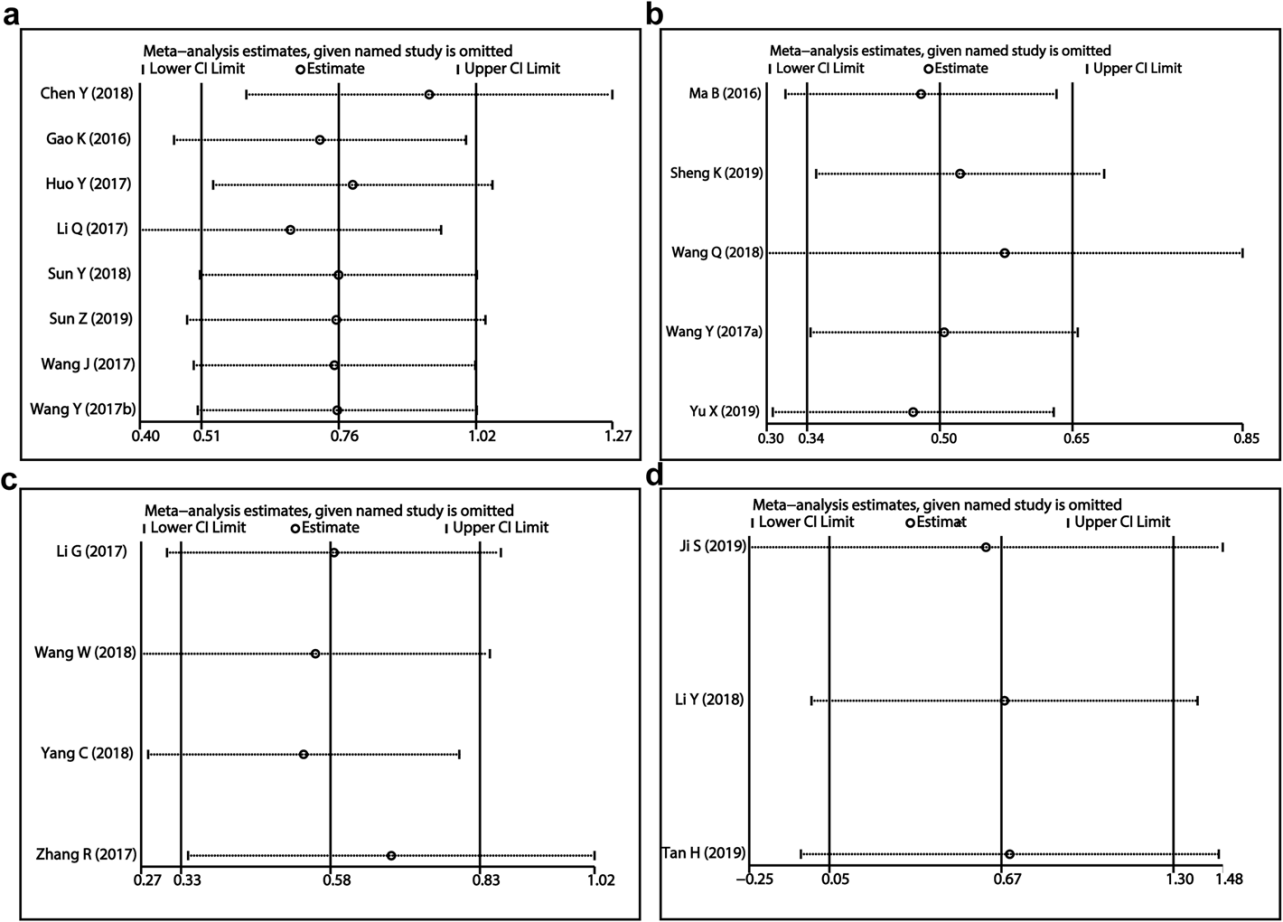
Sensitive analysis of lncRNAs for osteosarcoma: **a** MALAT1, **b** TUG1, **c** XIST, **d** NEAT1

We did sensitivity analysis to the four lncRNAs which had studied in more than three articles respectively even though there was no heterogeneity detected (I^2^ = 0.0%, *P* > 0.05). The results showed that this meta-analysis was reliable (Fig. 3).

### Publication Bias

**Fig. 4:**
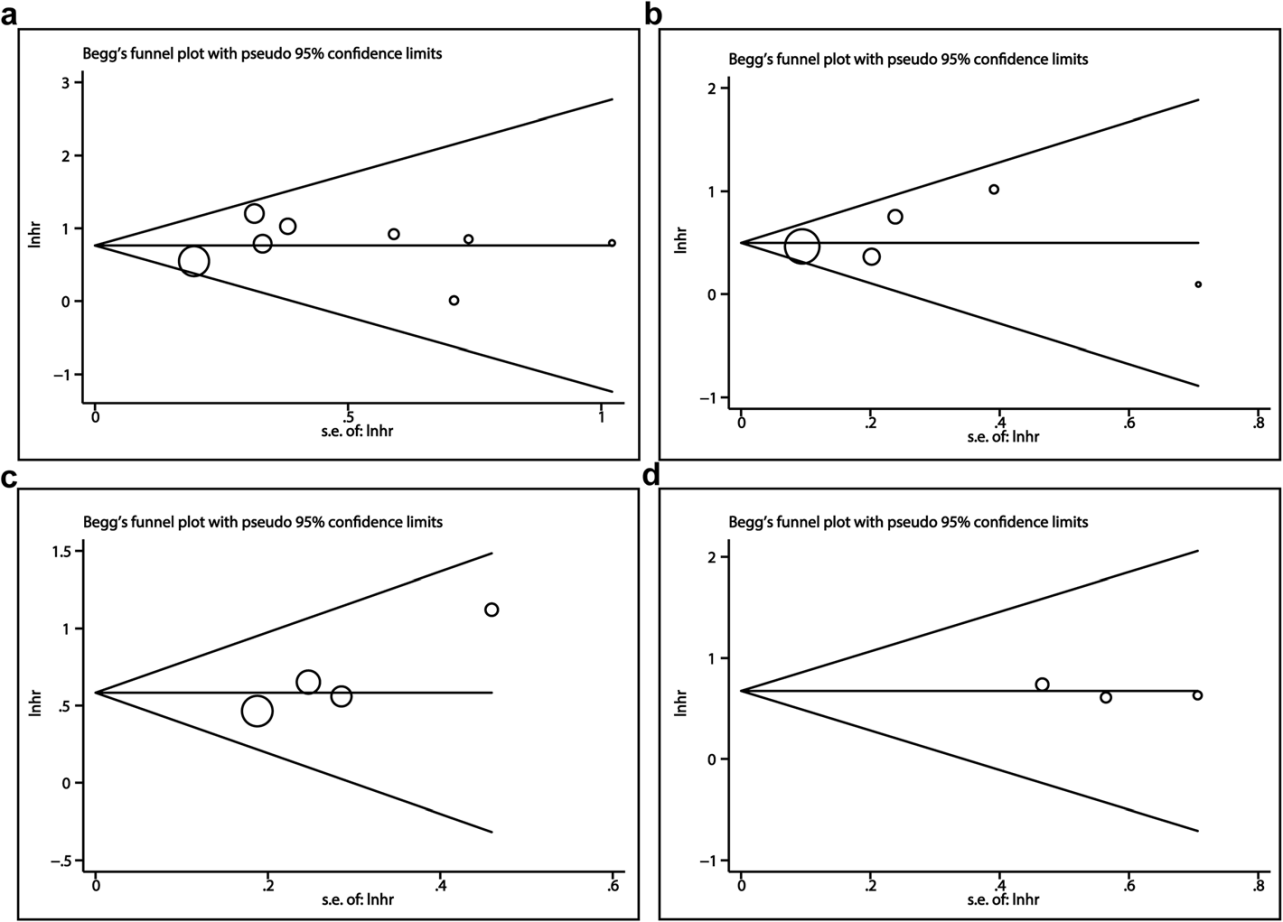
Funnel plots of the four most studied lncRNAs: **a** MALAT1, **b** TUG1, **c** XIST, **d** NEAT1

Publication bias was measured by using the Begg’s and Egger’s test. We only analyzed the publication bias of lncRNAs that have been studied in more than three articles, including MALAT1, TUG1, XIST and NEAT1. No significant publication bias was found in any of the lncRNAs. Begg’s funnel plot was shown in Fig. 4. However, publication bias between different lncRNAs was subsistent since the number of published articles lacked consistence for them.

## Discussion

Osteosarcoma remains intractable in clinical practice, and new approaches for prognostic evaluation and treatment of osteosarcoma are continuously requisite. Recently, targeted therapy and molecular biomarker diagnosis have emerged as the focus in cancers [82, 83]. LncRNAs, as indispensable regulators in a majority of biological processes [84], possess great potential for prognostic hallmarks. Further, advancement of technologies for structural and functional study enable us to unveil more evident features of lncRNAs serving as idea clinical biomarkers [85]. Considering the vast lncRNAs studied in osteosarcoma [14], we conducted the meta-analysis, with the aim to provide stronger evidences in this regard.

**Fig. 5:**
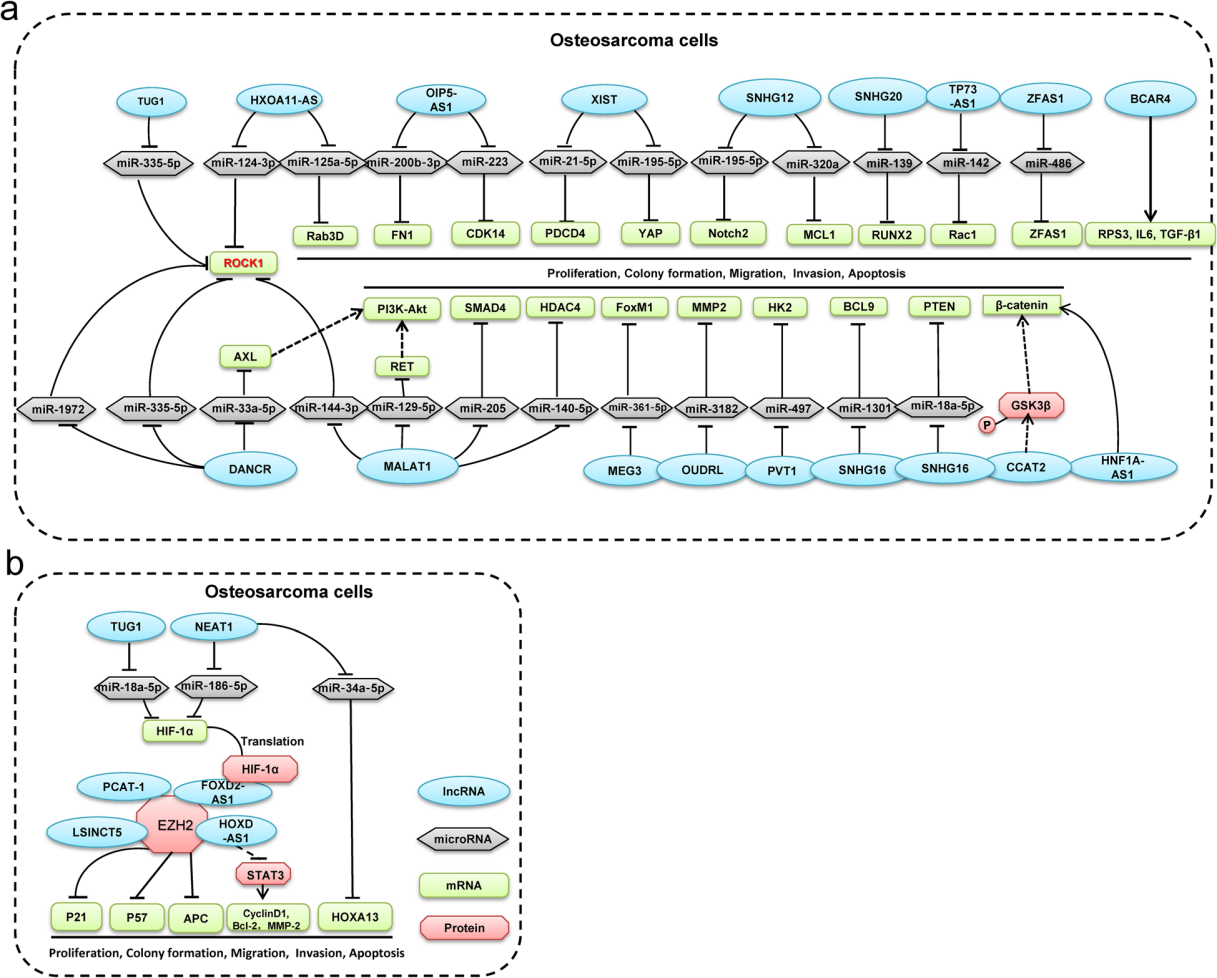
Schematic diagram of lncRNAs mechanism in osteosarcoma cells

In this meta-analysis, a total of 4351 cases were included, and 25 lncRNAs were analyzed in which high expression of 14 lncRNAs connotes worse OS while two were associated with positive outcomes. Mechanisms involved in these lncRNAs are multifaced. BCAR4 promoted proliferation and migration by GLI2 target genes including RPS3, IL6, MUC5AC and TGF-β [20, 21]. HNF1A-AS1 targeted Wnt/β-catenin pathway to enhance proliferation and G1/S transition, migration and invasion by reducing the EMT [31]. Meanwhile, MALAT1 positively regulated RET to activate the PI3K-Akt signaling pathway by competitively binding with miR-129-5p, and thus enhancing stem cell-like properties [40]. Furthermore, MALAT1/miR-144-3p/ ROCK1 axis promoted the proliferation and metastasis of osteosarcoma [40]. Moreover, MALAT1 promoted proliferation and metastasis via miR-205/SMAD4 axis [43] and miR-140-5p/HDAC4 axis [44]. NEAT1 could up-regulate HOXA13 by decoying of miR-34a-5p, while NEAT1/ miR-186-5p/HIF-1α axis enhanced proliferation and reduced apoptosis [50–52]. Rho-associated protein kinase 1 (ROCK1), a serine/threonine kinase, is critical regulator of development and progression in various human malignant tumors. Importantly, TUG1 served as a ceRNA of miR-335-5p to affect ROCK1-mediated migration and invasion [72]. Besides, other important hallmarks of osteosarcoma demonstrate close association with TUG1. The effects of TUG1 overexpression on runt-related transcription factor 2 (RUNX2) expression were elucidated. It was noticed that overexpression of lncRNA TUG1 significantly down-regulated RUNX2 level [70]. Likewise, TUG1 could impede osteosarcoma cells proliferation, migration, and invasion by miR-140-5p/PFN2 axis [86]. XIST is another potential biomarker of osteosarcoma which has been reported to modulate osteosarcoma proliferation and invasion through miR-320b/RAP2B [87], miR-193a-3p/RSF1 [88], miR-21-5p/PDCD4 [79], and miR-195-5p/YAP axis [78]. In addition, SNHG16/miR-1301/BCL9 axis [64], MEG3/miR-361-5p/FoxM1 axis [48], SNHG20/miR-139/RUNX2 axis [65], TP73-AS1/miR-142/Rac1 axis [68] and SNHG12/miR-195-5p/Notch2 [62] axis worked as critical roles of enhancing proliferation, migration and invasion. Additionally, OIP5-AS1 and SNHG12 were involved in osteosarcoma doxorubicin resistance via miR-200b-3p/FN1 and miR-320a/MCL1 pathways, respectively [53, 61]. Further, enhancer of zeste homolog 2 (EZH2) was involved in DNA methylation and its mutations have been identified in various malignancies. HOXD-AS1 suppressed p57 expression by binding with EZH2 [34]. LSINCT5 binding with EZH2 inhibited APC transcription that could down-regulate the Wnt/ β-catenin pathway and activate the PI3K-Akt signaling pathway [39]. The detailed mechanisms are shown in Fig. 5.

Previously, meta-analysis by Wang Y et al. in 2017 [47] and Chen D et al. in 2018 [89] have illustrated the relationship between osteosarcoma and lncRNAs. However, numbered lncRNAs (TUG1, UCA1, BCAR4, HULC, etc.) were analyzed, which led to significant limitation for their research. Among the 25 enrolled lncRNAs, four (MALAT1, TUG1, XIST and NEAT1) reported in more than three studies respectively have been the focus of our meta-analysis because efficacy confirmed in multiple datasets tend to be more convictive. Their high expression predicted poor prognosis of osteosarcoma (MALAT1 (HR = 2.15, 95%CI: 1.67–2.76, *P* < 0.001), TUG1 ((HR = 2.41, 95%CI: 1.42–4.07, *P* = 0.001), XIST (HR = 1.79, 95%CI: 1.40–2.30, *P* < 0.001), NEAT1 (HR = 1.96, 95%CI: 1.05–3.68, *P* = 0.035)). Specifically, we observed that, for lncRNA MALAT1 and TUG1, each contained one study that did not employ neoplastic tissue as the test item. Therefore, we did another analysis after eliminated them respectively in order to minimize the potential bias. Results showed no obvious difference compared to the previous analysis.

Besides, we have evaluated the relationship between lncRNAs expression and clinicopathological features of osteosarcoma. MALAT1 expression level was not associated with the age, gender, clinical stage, tumor size and metastasis. However, patients with elder age, larger tumor size and distant metastasis were accompanied by overexpression of TUG1 and XIST, which further demonstrated the negative role of lncRNA TUG1 and XIST in osteosarcoma progression. Furthermore, a series of lncRNAs have been elucidated to serve as important prognostic hallmarks in numerous tumors, for instance, MALAT1 in breast cancer and digestive system cancer [90, 91], XIST in various solid tumors [92], BCAR4 and SNHG16 in diverse human neoplasms [93–95].

To date, functional implications that support the prognostic roles of LncRNAs in human cancers have been expounded. Importantly, lncRNAs are capable of altering gene expression of cancer stem cells via interplaying with chromatin modification, transcriptional and post-transcriptional factors [96]. Cancer stem cells are critical initiators of tumors which are able to differentiate into heterogeneous lineages of cancer cells, thereby it is of great significance for neoplastic progress. Moreover, epithelial-mesenchymal transition, a prevalent process in tumors, is largely regulated by multiple lncRNAs transcriptionally or post-transcriptionally [84]. Besides, involvement of lncRNAs in regulating some key oncogenic factors such as p53 and MYC has provided evidence for their cancer-relevant functions [10]. And currently, the use of antisense oligonucleotides, small molecules for the targeting of lncRNAs, and tools based on CRISPR–Cas systems may provide new approaches for lncRNA-based targeted therapy [10]. However, adopting lncRNAs as the prognostic or therapeutic markers remains experimentally proposed since the lack of large sample trial to confirm their efficacy and safety. Our meta-analysis that pool and analyze the published dataset thus provide stronger evidence and somewhat promote the progress in this regard.

Ultimately, this meta-analysis yielded valuable results, but there were limitations: (1) Using different methods to extract data can lead to bias, and some HR values are obtained through the tool software indirectly, which makes the bias even greater. (2) Almost all of the included studies are from China, leading to bias caused by geographical differences. (3) Some enrolled studies have different follow-up time and cut-off value.

## Conclusions

In conclusion, our study confirmed that lncRNAs are of significant potential in serving as molecular markers for prognosis of osteosarcoma. High expression of a set of lncRNAs predict positive prognosis while some indicate poor outcomes. This meta-analysis has laid a theoretical foundation for experimental exploration and clinical application of lncRNAs in the future.

## Supplementary Information

**Additional file 1 **Study quality and bias in the retrospective cohort studies judged by the Newcastle-Ottawa Scale (NOS) checklist. **Figure A.1** Quality assessment of all included studies. “Risk of bias summary” of all included studies. **Figure A.2** Quality assessment of all included studies. “Risk of bias graph” of all included studies.

**Additional file 2.** PRISMA Checklist.

## Data Availability

The datasets used and/or analysed during the current study are available from the corresponding author on reasonable request.

## Abbreviations

LncRNA: Long non-coding RNA
OS: overall survival
HR: hazard ratio
NOS: Newcastle-Ottawa Scale
MALAT1: Metastasis-associated lung adenocarcinoma transcript 1
TUG1: Taurine up-regulated gene 1
XIST: X-inactive specific transcript
NEAT1: nuclear enriched abundant transcript 1
ROCK1: Rho-associated protein kinase 1
EZH2: Enhancer of zeste homolog 2
