# Supplementary figures and images for: The value of lncRNAs as prognostic biomarkers on clinical outcomes in osteosarcoma: a meta-analysis
Source: BMC Cancer. 2021 Feb 27;21:202. doi: 10.1186/s12885-021-07882-w (PMC7912917; doi:10.1186/s12885-021-07882-w)

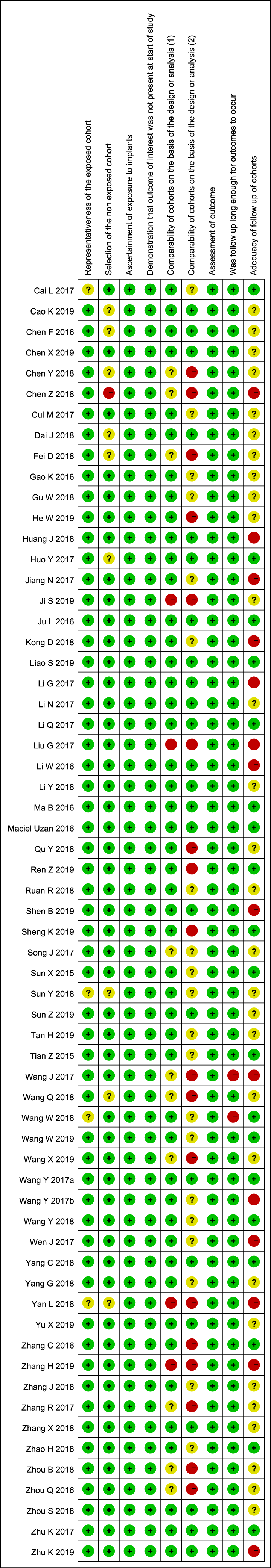

Supplement: Supplementary file 1 — Additional file 1 Study quality and bias in the retrospective cohort studies judged by the Newcastle-Ottawa Scale (NOS) checklist. Figure A.1 Quality assessment of all included studies. “Risk of bias summary” of all included studies. Figure A.2 Quality assessment of all included studies. “Risk of bias graph” of all included studies. [file 12885_2021_7882_MOESM1_ESM.zip › Additional figure S1R2.tif]

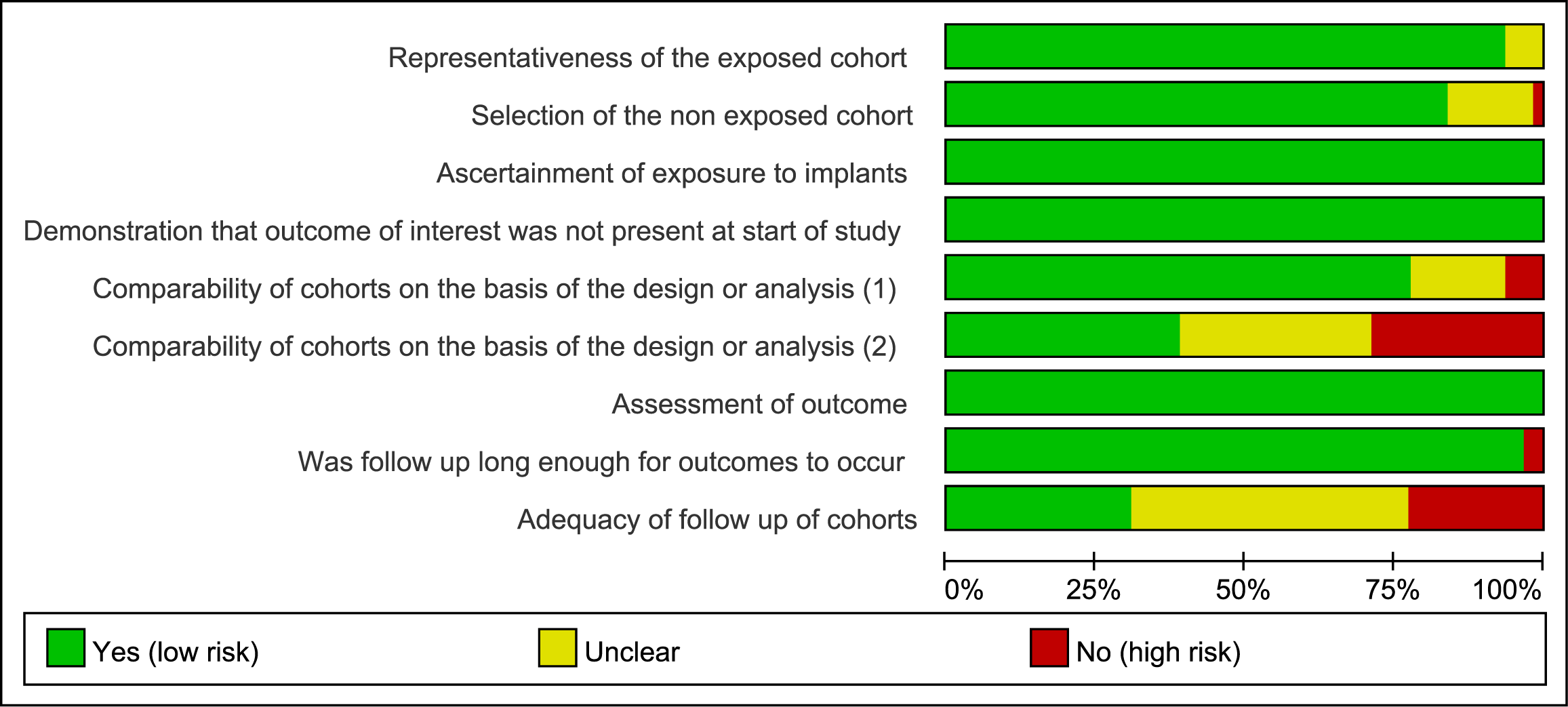

Supplement: Supplementary file 1 — Additional file 1 Study quality and bias in the retrospective cohort studies judged by the Newcastle-Ottawa Scale (NOS) checklist. Figure A.1 Quality assessment of all included studies. “Risk of bias summary” of all included studies. Figure A.2 Quality assessment of all included studies. “Risk of bias graph” of all included studies. [file 12885_2021_7882_MOESM1_ESM.zip › Additional figure S2R2.tif]
